# ‘We were only allowed to perform an autopsy on those patients we had taken good care of’

**DOI:** 10.1098/rstb.2008.4020

**Published:** 2008-11-27

**Authors:** Pako Ombeya

**Affiliations:** Waisa Village, c/o Papua New Guinea Institute of Medical ResearchPO Box 60, Goroka, EHP 441, Papua New Guinea

Today, I am so happy to see some of the colleagues whom I once worked with in Okapa. I would like to take this opportunity to thank all the medical scientists who came to work on kuru. Back in my village of Waisa, I was newly married when Michael Alpers arrived in our village to carry out his field research. The kuru epidemic was frightening and taking the lives of many women and children and also men. The worst affected villages came to a stage where there were many orphans to care for and not enough women for men to marry.

Michael fitted in well with my family. In fact, all the village people regarded Michael as one of our family members. We gave him a block of land and built him a house to settle down in and work.

I mainly assisted Michael in fieldwork on kuru and explained to the people why he was there and what he wanted to do. In the minds of the people, they feared that kuru was caused by sorcery and the collection of any samples from humans was very hard. The fear of sorcery was very high but we had a good team and it worked out well. Dead bodies were also highly respected in the South Fore and getting permission to perform an autopsy on dead patients was hard. We were only allowed to perform an autopsy on those patients we had taken good care of.

